# RETRACTION: A Retina‐Inspired Optoelectronic Synapse Using Quantum Dots for Neuromorphic Photostimulation of Neurons

**DOI:** 10.1002/advs.75409

**Published:** 2026-04-20

R. Balamur, G. O. Eren, H. N. Kaleli, O. Karatum, L. Kaya, M. Hasanreisoglu, and S. Nizamoglu, “A Retina‐Inspired Optoelectronic Synapse Using Quantum Dots for Neuromorphic Photostimulation of Neurons,” *Advanced Science* 11, no. 20 (2024): 2306097, https://doi.org/10.1002/advs.202306097.

The above article, published online on 21 March 2024 in Wiley Online Library (wileyonlinelibrary.com), has been retracted by agreement between the journal Editor‐in‐Chief, Kirsten Severing; and Wiley‐VCH GmbH. The retraction has been agreed due to an error at the publishers which caused this duplicate to be published. The correct version of the article was published on 06 March 2024 and is to be found at: R. Balamur, G. O. Eren, H. N. Kaleli, O. Karatum, L. Kaya, M. Hasanreisoglu, and S. Nizamoglu, “A Retina‐Inspired Optoelectronic Synapse Using Quantum Dots for Neuromorphic Photostimulation of Neurons,” *Advanced Science* 11, no. 18 (2024): 2401753, https://advanced.onlinelibrary.wiley.com/doi/10.1002/advs.202401753.

